# Supplementary material for: A Fungicide, Fludioxonil, Formed the Polyploid Giant Cancer Cells and Induced Metastasis and Stemness in MDA-MB-231 Triple-Negative Breast Cancer Cells
Source: Int J Mol Sci. 2024 Aug 20;25(16):9024. doi: 10.3390/ijms25169024 (PMC11354328; doi:10.3390/ijms25169024)
Supplement: Supplementary file 1 [file ijms-25-09024-s001.zip › Supplementary Figure S1.pptx]

## Slide 1
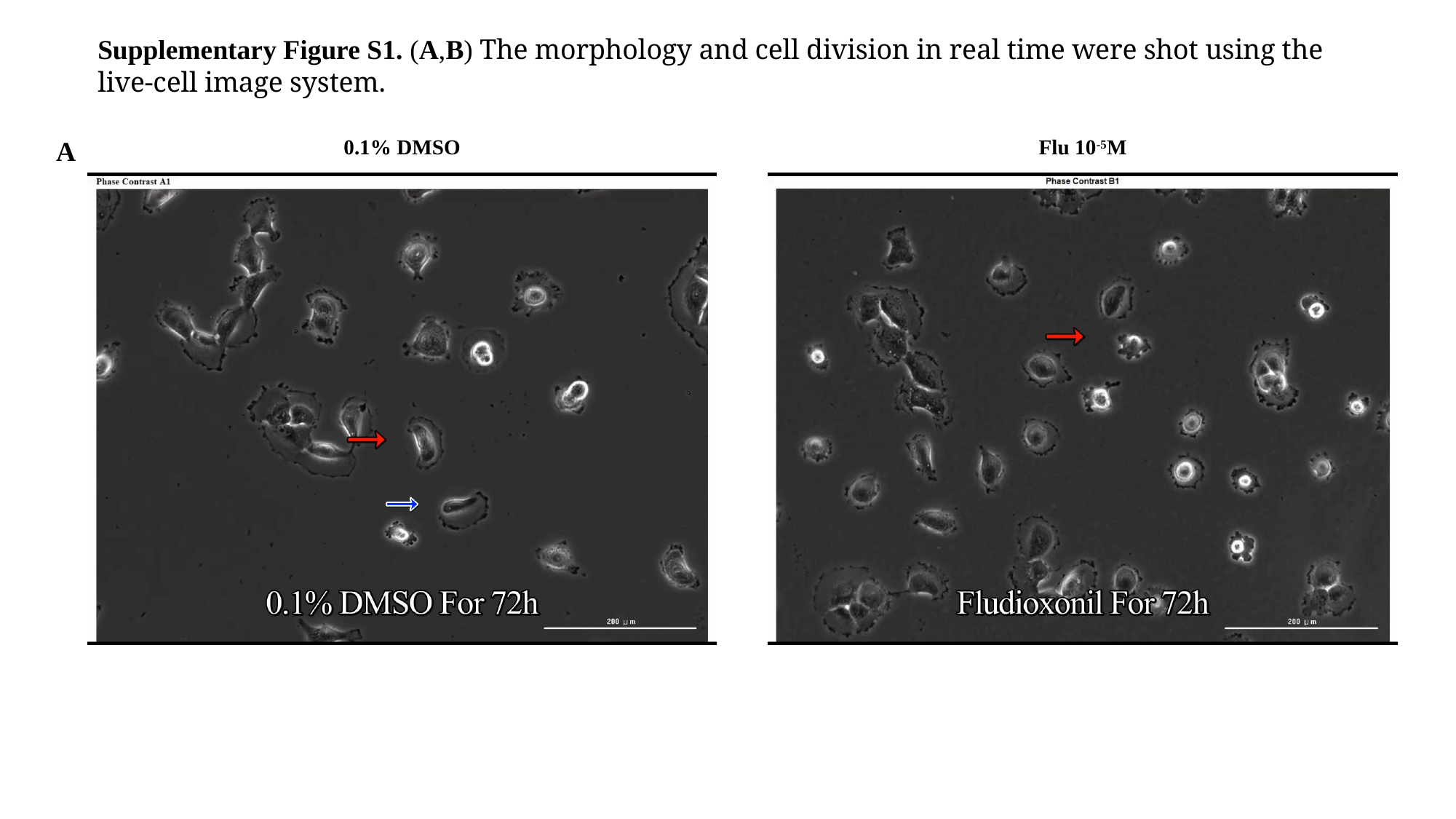

Supplementary Figure S1. (A,B) The morphology and cell division in real time were shot using the live-cell image system.
0.1% DMSO
A
Flu 10-5M
