# Supplementary material for: A Fungicide, Fludioxonil, Formed the Polyploid Giant Cancer Cells and Induced Metastasis and Stemness in MDA-MB-231 Triple-Negative Breast Cancer Cells
Source: Int J Mol Sci. 2024 Aug 20;25(16):9024. doi: 10.3390/ijms25169024 (PMC11354328; doi:10.3390/ijms25169024)
Supplement: Supplementary file 1 [file ijms-25-09024-s001.zip › Supplementary Table S1.pdf]

Supplementary Table S1. List of DEGs.

Gene	log2FC	*q-value*(FDR)	Gene	log2FC	*q-value*(FDR)
DHRS2	3.471	1.60E-04	SERPINB2	1.653	1.60E-04
ETV7	2.815	1.60E-04	LINC00923	1.630	6.87E-03
SLC12A7	2.466	1.60E-04	NR4A3	1.626	1.60E-04
DMC1	2.458	6.24E-02	MMP10	1.624	3.11E-04
ARC	2.375	1.60E-04	CSF3	1.620	1.60E-04
LINC00173	2.346	5.14E-03	SCEL	1.619	1.60E-04
LCN2	2.316	5.50E-03	GDF15	1.616	1.60E-04
S100P	2.280	1.60E-04	ATF3	1.608	1.60E-04
HIST1H2BN	2.265	4.43E-03	GPRC5B	1.602	1.60E-04
SLC22A1	2.262	1.60E-04	S100A14	1.564	3.36E-02
TNF	2.208	3.11E-04	TNNC1	1.562	8.77E-02
KRTAP4-9	2.096	5.51E-02	TRIM54	1.545	5.14E-03
CCL20	2.028	1.60E-04	ESM1	1.541	1.60E-04
NEURL3	1.956	8.57E-03	IL1B	1.537	1.60E-04
GALR2	1.914	2.20E-03	LCK	1.535	1.60E-04
LOC101927919	1.902	1.29E-02	ARL14	1.523	1.60E-04
LINC00622	1.894	1.02E-02	ELF3	1.508	1.60E-04
ALOXE3	1.888	1.60E-04	LST1	1.508	2.30E-02
PYCARD-AS1	1.886	6.02E-02	TNFRSF9	1.496	1.60E-04
MMP13	1.879	7.41E-04	ID2	1.489	1.60E-04
CEACAM1	1.854	5.03E-03	AADACP1	1.477	1.60E-04
CARD9	1.830	4.58E-04	KISS1	1.455	1.60E-04
DUSP8	1.785	1.60E-04	LINC01204	1.452	1.60E-04
PLAC1	1.765	8.01E-03	RARRES3	1.450	3.02E-02
AADAC	1.759	2.33E-03	INHBA	1.450	1.60E-04
MYPN	1.724	1.60E-04	LRG1	1.424	1.60E-04
DHDH	1.719	4.84E-02	HIST1H4H	1.420	1.60E-04
C1orf162	1.692	8.80E-04	IL1A	1.419	1.60E-04
LINC00471	1.688	1.86E-02	UFSP1	1.408	1.59E-02
SAA2	1.674	4.33E-02	HIST1H2AC	1.398	1.60E-04
TRIM55	1.670	1.60E-04	GBP5	1.397	1.60E-04
RAET1L	1.669	4.19E-03	TCN1	1.393	7.41E-04
LINC01556	1.669	6.56E-02	TXNIP	1.387	5.73E-03

FAM86HP	1.375	7.34E-03	IL15RA	1.234	1.60E-04
OLAH	1.371	1.15E-02	AREG	1.230	1.60E-04
MATN4	1.368	2.20E-03	HIST1H2BJ	1.224	1.60E-04
EGR2	1.359	1.60E-04	DHRS3	1.219	1.60E-04
LOC101927934	1.356	6.08E-03	PI3	1.214	6.62E-02
PDCL3P4	1.356	6.19E-02	EGR1	1.207	1.60E-04
PTPRH	1.349	1.60E-04	HIST1H1C	1.206	1.60E-04
FLJ27354	1.335	5.36E-02	LINC01119	1.202	4.43E-03
L1CAM	1.334	1.60E-04	HSPA6	1.198	1.60E-04
SLC16A1	1.327	1.60E-04	ZBED6CL	1.195	1.60E-04
C10orf54	1.326	1.60E-04	SPX	1.184	4.03E-02
BTG2	1.317	1.60E-04	C9orf84	1.183	1.60E-04
RPLP0P2	1.317	1.60E-04	LOC100289361	1.177	2.66E-02
FCMR	1.316	1.60E-04	LOC653712	1.174	4.79E-03
LOC730202	1.309	5.57E-02	FAM27B	1.172	1.60E-04
SBSN	1.307	4.43E-03	MUC20	1.171	8.57E-03
CLDN1	1.305	1.60E-04	MIA2	1.168	4.58E-04
MMP3	1.303	7.53E-02	LINC01186	1.162	3.59E-02
LOC101929715	1.295	4.15E-02	MB	1.157	5.18E-02
CH25H	1.294	7.41E-04	IL7R	1.156	1.60E-04
TMEM139	1.292	1.60E-04	PLA2G4C	1.153	1.60E-04
KRT17P5	1.291	1.60E-04	CXCL3	1.153	1.60E-04
BIK	1.290	1.66E-02	TRAF1	1.148	1.60E-04
IL2RG	1.287	3.96E-02	C11orf86	1.143	1.60E-04
FOSB	1.279	1.60E-04	KRTAP4-8	1.142	2.08E-03
ARRDC4	1.274	1.60E-04	PTPRR	1.136	1.60E-04
LOC100996286	1.270	1.42E-03	LINC01144	1.135	4.67E-03
EBI3	1.268	4.14E-02	HIST1H2BD	1.135	1.60E-04
SH2D1B	1.260	1.60E-04	IL6	1.127	1.60E-04
LOC101927765	1.252	5.77E-02	UBE2Q2L	1.124	6.45E-02
FES	1.248	1.29E-03	LOC100129917	1.123	1.60E-04
C15orf48	1.248	1.60E-04	MAL2	1.117	1.60E-04
CPLX3	1.241	3.34E-03	C8orf37-AS1	1.114	6.33E-02

IL23A	1.112	1.60E-04	PYGM	1.063	1.60E-04
AKNAD1	1.112	1.60E-04	XIRP2	1.054	3.11E-04
SCG2	1.109	1.60E-04	GPNMB	1.053	4.58E-04
PGM5	1.107	3.09E-03	DDIT3	1.048	4.58E-04
DMKN	1.106	1.60E-04	HKDC1	1.041	1.60E-04
NR4A1	1.095	1.60E-04	LOXL4	1.041	1.60E-04
AQP3	1.095	1.60E-04	STC2	1.037	1.60E-04
OSGEPL1-AS1	1.094	8.05E-02	PTGS2	1.037	1.60E-04
CNKSR1	1.093	3.46E-03	LOC100505817	1.036	3.11E-04
RND1	1.091	1.60E-04	AGR2	1.026	9.35E-03
RRAD	1.090	1.60E-04	BMP2	1.025	1.60E-04
AOC2	1.090	1.60E-04	MAP3K7CL	1.018	1.60E-04
SNAI1	1.090	1.60E-04	HIST1H2BC	1.016	1.60E-04
IL32	1.087	1.60E-04	ICAM1	1.015	1.60E-04
LOC100506860	1.086	3.46E-03	AOC3	1.010	1.60E-04
TOLLIP-AS1	1.082	2.87E-02	NEAT1	1.006	1.60E-04
CCL26	1.081	2.20E-03	LOC101929295	1.006	1.58E-02
LOC100272217	1.078	1.60E-04	ST6GALNAC5	1.005	3.11E-04
NGF	1.073	8.01E-03	LINC01589	1.003	3.80E-02
CHKB-AS1	1.071	3.17E-02	ACAP1	1.001	1.56E-03
TM4SF19-TCTEX1D2	1.069	1.05E-02	-	-	-

FC: fold change, FDR: false discovery rate.
